# Synthesis of ursodeoxycholic acid by electrochemical stereoselective reduction of 7-ketolithocholic acid in aprotic solvents

**DOI:** 10.1038/s41598-021-95577-4

**Published:** 2021-08-11

**Authors:** Jinxue Shen, Dongdong Dong, Zefa Wang, Junfen Wan, Xuejun Cao

**Affiliations:** State Key Laboratory of Bioreactor Engineering, Department of Bioengineering, East China University of Science and Technology, Shanghai, 200237 China

**Keywords:** Chemical biology, Chemistry

## Abstract

A novel method of producing ursodeoxycholic acid was developed through electrochemical stereoselective reduction of 7-ketolithocholic acid (7K-LCA) in a undivided electrolytic cell and aprotic solvents as electrolyte. Five aprotic solvents were investigated as electrolytes, the simple structure of dimethyl sulfoxide (DMSO) and *N,N*-dimethylformamide (DMF) were easily attacked by chloride ions and undergo nucleophilic reactions, resulting in no target reactions. The structure of hexamethylphosphoric triamide (HMPA) and 1,3-methyl-3,4,5,6-tetrahydro-2 (1H) -pyrimidinone (DMPU) is relatively complex, but chloride ions can still attack them, and it was easier for 7K-LCA to directly undergo a reduction reaction under the action of electric current, because of the small steric hindrance of chenodeoxycholic acid (CDCA), 7K-LCA was stereoselectively reduced to CDCA. Due to the stable structure of the five-membered imidazole ring of 1,3-dimethyl-2-imidazolidinone (DMI), 7K-LCA undergoes two nucleophilic and a "Walden inversion", thereby stereoselective reduction of 7K-LCA to UDCA. In DMI, the highest conversion rate of 7K-LCA was 58.3%, the yield of UDCA was 34.9%, ee value was 100%. Linear sweep voltammetry was used to explore the electrochemical behavior of the reaction, and the electrolysis results were consistent with the linear sweep voltammetry. The product was characterized by using IR, ^1^H NMR and ^13^C NMR, it confirm the product was UDCA. The method developed in this paper provides a relatively environmentally friendly and low-consumption method for large-scale production of ursodeoxycholic acid, and provides a valuable reference for the asymmetric electrochemical reduction of ketone groups.

## Introduction

**Figure 1:**
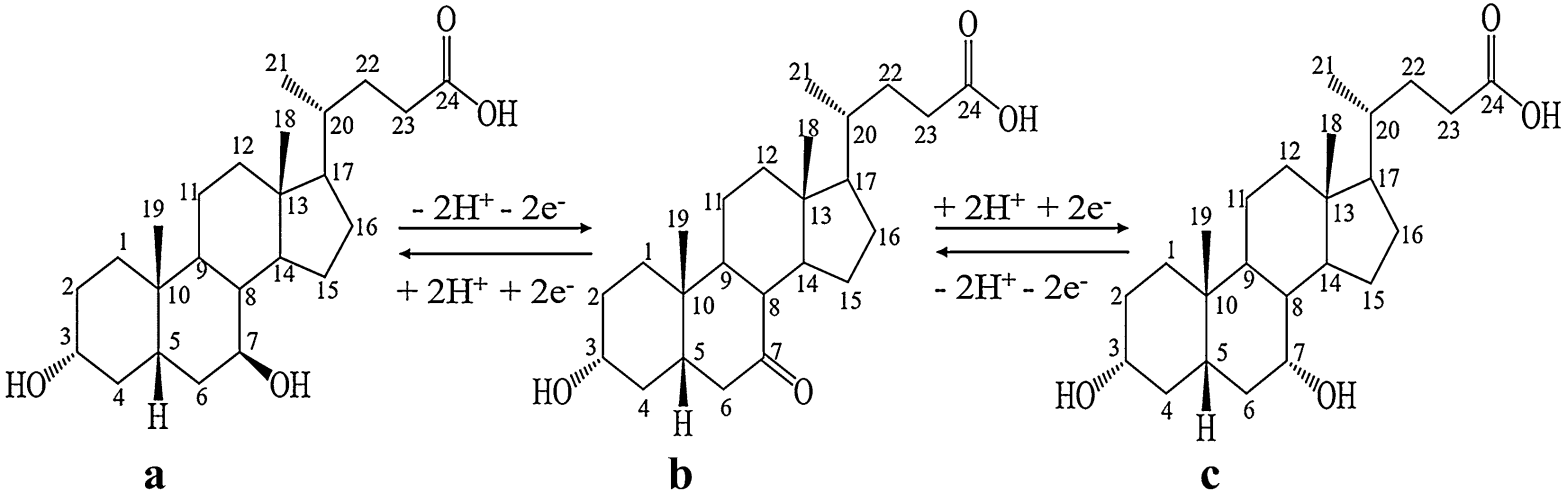
The chemical structure of UDCA **(a)**, 7K-LCA **(b)** and CDCA **(c)**.

Ursodeoxycholic acid (3α, 7β-2-hydroxy-5β-cholanic acid, UDCA,) was a secondary bile acid and was an important component in the precious bile of Chinese herbal medicine. Because ursodeoxycholic acid was an important clinical drug for the treatment of certain diseases, such as primary biliary cirrhosis, primary sclerosing cholangitis, gallstones, viral hepatitis, alcoholic fatty liver disease, non- alcoholic fatty liver disease and rectal cancer^1–4^, so it has high medicinal value and significance for in-depth research. The initial method for obtaining ursodeoxycholic acid was to arrest wild bears and extract bile. Or artificially rearing bears and with the bear alive, insert a catheter to collect the bear bile to extract ursodeoxycholic acid. However, considering that bears were protected animals in the world, it was illegal using these methods to obtain ursodeoxycholic acid. Therefore, the efficient, fast and economical method of artificially synthesizing ursodeoxycholic acid has been studied in many aspects by scholars in the chemical and biological industries. Most scholars used raw materials that were similar in structure to UDCA and were easily available. Chenodeoxycholic acid (CDCA) was derived from chicken bile and its only hydroxyl conformation at the C7 position was different from that of UDCA, so CDCA had become the first choice of many researchers. But it was difficult to directly change the conformation of the hydroxyl group convert from CDCA to UDCA, so the researchers first oxidized the hydroxyl group at the C7 of CDCA to a ketone group to obtain 7-ketolithocholic acid (7K-LCA), and then reduced to obtain UDCA and CDCA in a ratio of 1:1, theoretically (Fig. 1).

In 1950s, Kanazawa et al.^5^ first reported that ursodeoxycholic acid was produced by reducing 7K-LCA in acetone using sodium metal. After that, other scholars also studied the use of alkali metals to reduced 7K-LCA, but the use of alkali metals in the reaction process was dangerous and uncontrollable. The safety performance requirements of reaction equipment were also improved at the same time. In 2020, Jie Wang^6^ used plant-derived (20S)-21 hydroxy-20-methylpregn-4-en-3-one as the initial substrate and synthesized ursodeoxycholic acid through a six-step chemical reaction without alkali metals, the final yield of UDCA was 59%. It can be seen that after the alkali metal was not used in the reaction, the steps of the chemical synthesis method complicated, which will also lead to an increase in the cost of production.

In order to find a better synthesis method, there were researchers studying the use of biological methods to synthesize ursodeoxycholic acid. In 2015, Zheng Mingmin^7^ discovered a new 7-hydroxysteroid dehydrogenase in Ruminococcus torques, combined with another NADPH-dependent 7α-HSDH from Clostridium, used a one-step method to convert CDCA to UDCA, the yield was 73%, used a two-step method to first convert CDCA to 7K-LCA, and then convert 7K-LCA to UDCA, the yield increased to 98%. Later in 2017, he^8^ combined 7β-hydroxysteroid dehydrogenase and glucose dehydrogenase, and the yield of UDCA was close to 100%. Although the biosynthesis method had achieved high selectivity and yield, the enzyme will be inactivated during the reaction process and thus has a certain degree of instability.

**Figure 2:**
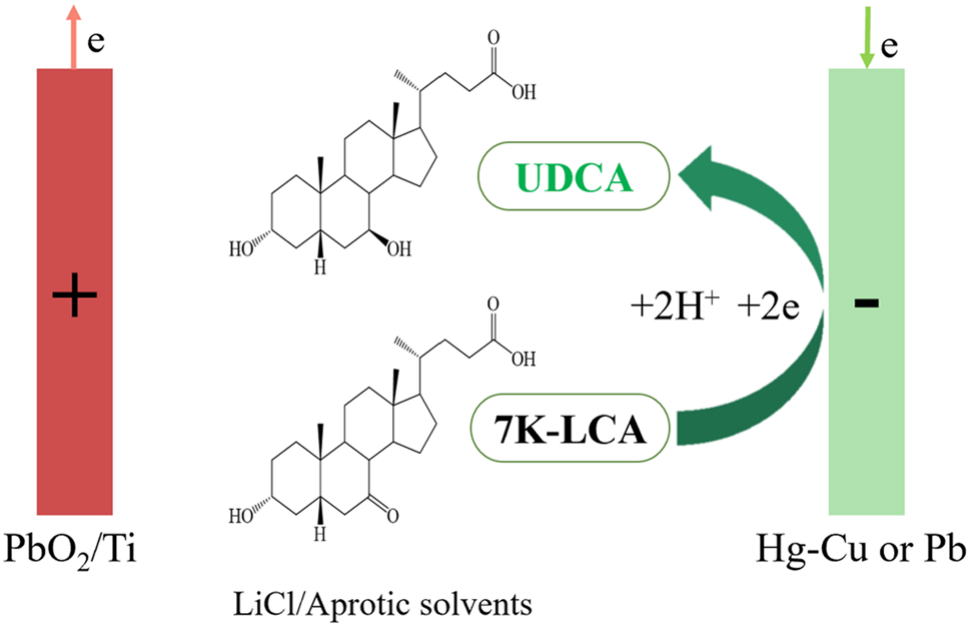
The reaction for electroreduction of 7K-LCA into UDCA in H-type electrolytic cell.

Compared with chemical and biological methods, there were relatively few studies on the electrochemical synthesis of ursodeoxycholic acid. In the 1980s, the method of electrochemical synthesis of ursodeoxycholic acid was discovered. In 1985, in the US patent^9^, 7K-LCA was electrochemically reduced to UDCA by using a mixture of lower alcohol and polar solvents as an electrolyte, the ratio of UDCA to CDCA in the product obtained by reduction as high as 5:1, but failed to avoid the formation of the enantiomer CDCA. In 1993, in the Japanese patent ^10^, 7-ketolithocholic acid was reduced to ursodeoxycholic acid by electrochemical reduction in short chain alcohol. In the previous work^11^, our research team successfully synthesized 7K-LCA by indirect electrooxidation of CDCA in short-chain alcohols, but when we wanted to reduced 7K-LCA in lower alcohols to obtain UDCA, we found that due to steric hindrance, 7K-LCA was easier to reduce to CDCA, and in an H-type electrolytic cell separated by a cation exchange membrane, methanol was used as the catholyte and sulfuric acid was used as the anolyte. It was found that a large amount of esterification (by-products a) were generated, there was a carboxyl group at C24 of 7K-LCA, it was very easy to undergo esterification reaction with methanol during the electrolysis process, which generates esterification, resulting in the UDCA cannot be generated. Similarly, the two hydrogen ions transferred to the cathode can also obtain electrons to generate hydrogen (by-products b), thereby competing with the reduction reaction for hydrogen ions, and the reduction reaction becomes more difficult to occur. (Fig. 2).

The existing electrochemical methods cannot achieve good stereoselectivity, but compared to the insecurity of chemical methods and the instability of biological reduction methods, the steps of electrochemical synthesis were simple, the operation was relatively safe, the reaction was stable, and the cost was low, which was very suitable for industrial production of UDCA. The electrochemical method was still worthy of further research and optimization. Therefore, in this paper, aprotic solvents were used as an electrolyte to prevent the esterification reaction. And some studies had shown that some aprotic solvents has certain stereoselectivity in the synthesis.

Hexamethylphosphoric triamide (HMPA)^12–15^ plays a key role in promoting many organic reactions mediated by organometallic catalysts or reagents. In some reduction reactions without adding HMPA, the carbonyl group cannot be reduced to the ketone radical anion, and the reaction time also need to be extended at a higher temperature. When HMPA was added, the ketone radical anion was more stable compared to no addition. It can also enhance the stereochemical results of the reaction and increase the reducing strength. But HMPA was highly carcinogenic. Some studies had shown that 1,3-methyl-3,4,5,6-tetrahydro-2 (1H) -pyrimidinone (DMPU) ^16,17^ also has a certain catalytic ability in the reduction reaction, and promoting the regio- and chemoselective reduction of aldehydes in orthoposition to phenols, it was stronger than HMPA, and can reduce the energy required for the reaction to occur. DMPU was considered as the best base in promoting the intramolecular hydride delivery. Some studies had shown 1,3-dimethyl-2-imidazolidinone (DMI)^18–20^ also was a special solvent to promote the reduction, but detail of the effect of DMI was still not clear at present. So DMI and DMPU can be used in place of HMPA in certain reactions.

The advantage of aprotic solvents is to promote the reaction at room temperature. However, some organic chemical synthesis reactions still need to be carried out under high or low temperature conditions. In this article, the electrochemical organic synthesis conditions were very mild. At normal temperature and pressure, DMI can be used as an electrolyte for stereoselective synthesis of ursodeoxycholic acid. When HMPA and DMPU were used as electrolyte, the results showed that CDCA was synthesized stereoselectively.

7K-LCA loses two electrons and two protons on the surface of the anode electrode and oxidizes to 3,7diketolithocholic acid. 7K-LCA gets two electrons and two protons on the surface of the cathode electrode and undergo a reduction reaction to produce UDCA or CDCA. At the same time, these two protons can also get two electrons on the cathode surface to generate hydrogen gas. The electrochemical reaction mechanism in undivided electrolytic cell in Fig. 1.

## Experimental

### Chemical reagent

The five aprotic polar solvents used in this study were dimethyl sulfoxide (DMSO, ≥ 99.0%), *N,N*-dimethylformamide (DMF, 99.5%), hexamethylphosphoramide (HMPA, 98%), 1,3-dimethyl-3,4,5,6-tetrahydro-2(1H)-pyrimidinone (DMPU, 99%) and dimethyl-2-imidazolidinone (DMI, 98%) from Shanghai Macklin Biochemical Technology Co. Ltd. In all solutions, anhydrous LiCl (Shanghai Aladdin Biochemical Technology Co., Ltd, 99%) was used as a supporting electrolyte, 7-ketolithocholic acid (7K-LCA, Anhui Kebao Biological Engineering Co., Ltd, 99%) as reducing substrate; Ursodeoxycholic acid (UDCA, 99%) and chenodeoxychol acid (CDCA, 99%) from Shanghai Macklin Biochemical Technology Co., Ltd; Mercury (Hg, 99%) from Shanghai Aladdin Biochemical Technology Co., Ltd; All other reagents were of analytical grade, from Sinopharm Chemical Reagent Co., Ltd.

### Instrumentation

Linear sweep voltammetry (LSV) experiments were carried out under nitrogen in a sealed three-electrode electrolytic cell at room temperature with a model CS150H electrochemical workstation (Wuhan CorrTest Instruments Co., Ltd.). The sealed three-electrode electrolytic cell was purchased from China Instrument No. 1 Store. Mercury-plated copper wire were used as working electrode (WE) of 1 mm diameter, the copper wire was purchased from Qinghe Saiwei Precision Metal Material Co., Ltd. The mercury-plated copper wire was made by ourself. The ruthenium-titanium mesh (S = 0.0009 m^2^, Henan Xinxiang Future Water Chemical Co., Ltd.) was used as the counter electrode (CE), and the saturated calomel electrode (SCE, Shanghai Yidian Scientific Instrument Co., Ltd.) was used as the reference electrode (RE).

Constant current electrolysis was carried out at room temperature with a Model HYD250-1 DC Stabilized Current Supply (Shanghai Huyi Technology Co., Ltd.). The copper (30 mm × 5 mm × 10 mm, 99.99%) and the lead (30 mm × 5 mm × 10 mm, 99.99%) were purchased from Safeway Precision Metal Materials Co., Ltd. The mercury plated copper (99.99%) plate made by ourselves or the lead as cathode material. The ruthenium titanium as anode material. Electrode materials were ultrasonically cleaned with dilute hydrochloric acid (Sinopharm Chemical Reagent Co., Ltd, 36%-38%) and ultrapure water before use and then polished (except ruthenium titanium). Keep all experimental materials dry before starting the experiment and all experiments were performed at room temperature under atmospheric pressure.

### Experimental procedure

Dissolved 0.25 g anhydrous LiCl in 60 mL DMSO, DMF, HMPA, DMPU and DMI, respectively. After completed dissolution, let the nitrogen passed through the solution for 10 min to removed oxygen that may affect the later test. After sealing, perform LSV test in this electrolyte. After the test, added 1.0 g 7K-LCA to the previous electrolyte separately. After dissolution, repeat the previous deoxygenation operation. After sealing, perform LSV test, and compare the linear sweep voltammograms obtained at different scan rates.

Constant current electrolysis was performed under the electrolysis system that dissolved 0.25 g anhydrous LiCl in 60 mL DMSO, DMF, HMPA, DMPU and DMI, respectively, after completed dissolution, added 1.0 g 7K-LCA. Current setting was 0.1 A. The electrolysis samples were taken every two hours during the electrolysis process, and the substrate conversion rate and product yield were detected by high-performance liquid chromatography. Increase the ion conductivity by increasing the LiCl concentration. The minimum concentration of support electrolyte was 0.1 M in the electrochemical experiment, dissolved 0.5 g and 0.75 g LiCl in HMPA, DMPU and DMI, respectively, and then dissolve 1 g 7K-LCA, respectively. The electrolysis time was the same as before.

### Analytical methods

Separation method: after the electrolysis was completed, a large amount of ultrapure water was added to the electrolytic solution and adjusted to acidity with HCl to precipitate the product. After filtration, the product was placed in a vacuum oven at 55 °C (Shanghai Yiheng Scientific Instrument Co., Ltd.) and dried for 12 h.

The products were analyzed by reversed phase HPLC, using a C-18 column with Welchrom-C18 5-μm resin in a 4.6 × 150 mm column (Welch Material Inc., Shanghai, China). The HPLC instrument (LC-20A) was from Shimadzu Corporation, Kyoto, Japan, and was used for quantitative analysis of the reaction products at 208 nm. The mobile phase was a mixture of acetonitrile and phosphate buffer (pH 2.0–3.0) with a volume ratio of 50:50 at a flow rate of 1.0 ml/min at 40 °C. Fifty percent methanol was used to clean the syringe in the SIL-20A autosampler (Shimadzu Corporation, Kyoto, Japan). The conversion of 7K-LCA was calculated as C = M_t_/M_0_ × 100%, where M_t_ was the reduction of 7K-LCA concentration and M_0_ was the total concentration of 7K-LCA before the electrolysis. The yield of UDCA was calculated as Y_u_ = U_a_/U_t_ × 100%, where U_a_ was the actual yield of UDCA and U_t_ was the theoretical yield of UDCA, U_t_ was equal to the total concentration of 7K-LCA before the electrolysis. The yield of CDCA was calculated as Y_c_ = C_a_/C_t_ × 100%, where C_a_ was the actual yield of CDCA and C_t_ was the theoretical yield of CDCA, C_t_ was equal to the total concentration of 7K-LCA before the electrolysis.

Thin layer chromatography to assist in the qualitative detection and analysis of the product, Thin layer chromatography pre-coated plates GF254 (Sinopharm Chemical Reagent Co., Ltd) as the chromatography plate. Spreading agent: prepare a certain volume of glacial acetic acid-acetone-dichloromethane solution, volume ratio was 1:10:20. Color developer: weigh a certain volume of concentrated sulfuric acid-glacial acetic acid (1:20) mixed solution, and then weigh the corresponding amount of phosphomolybdic acid solid to prepare a phosphomolybdic acid solution with a mass concentration of 4.5% as a color developer. Preparation of standard samples: prepare a water–acetone mixed solution with a volume ratio of 1: 9, then weigh 0.01 g of UDCA, CDCA and 7K-LCA standards, and dissolve them in 1 mL of water–acetone mixed solution to obtain a concentration of 10 mg/mL standard pure product solution. Preparation of product samples: Weigh 0.01 g of the product obtained after separation and drying at the end of electrolysis, and dissolve it in 1 mL of water–acetone mixture to make the concentration 10 mg/mL.

## Results and discussion

### Effects of different aprotic solvents

The linear sweep voltammograms tested in various systems were as follows.

**Figure 3:**
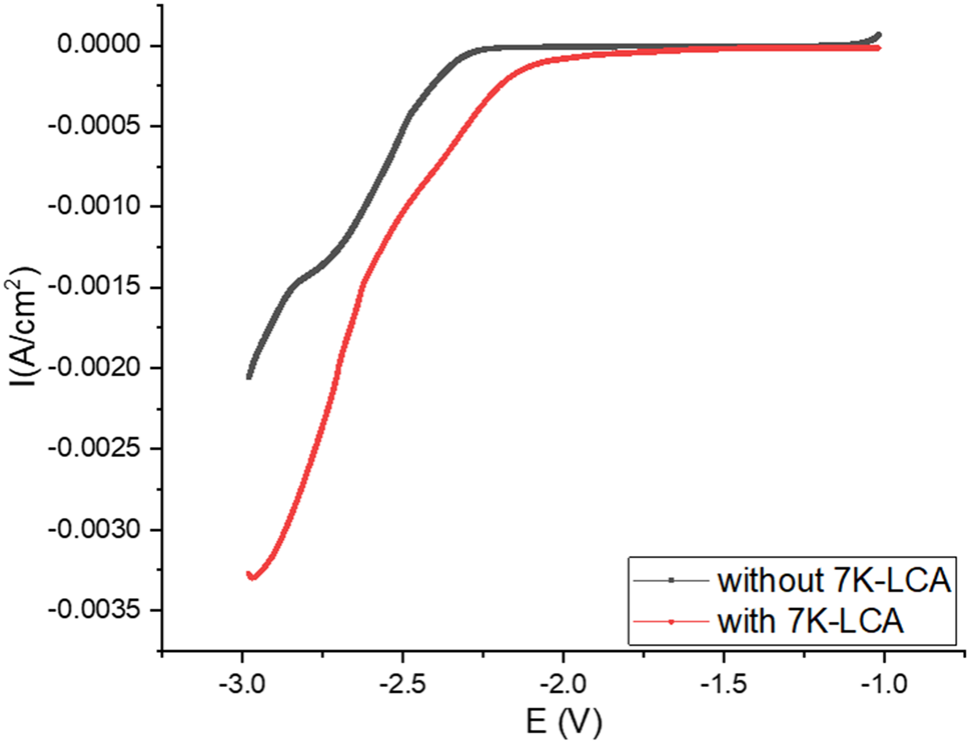
LSV of with and without 7K-LCA at 10 mv/s of scan rate (in DMSO).

As shown in Fig. 3, in the electrolyte system used DMSO as the solvent, the linear sweep voltammogram obtained by testing with and without 7K-LCA. It can be seen that in the electrolytic system without substrate, the measured linear sweep voltammogram showed a weak reduction peak, indicating that the solvent was reduced in this system. Also in an electrolytic system containing a substrate, the measured linear sweep voltammogram showed that the reduction peak of the solvent was negatively shifted due to the addition of the substrate, and no new reduction peak appears, indicating that 7K-LCA cannot be reduced in this system.

**Figure 4:**
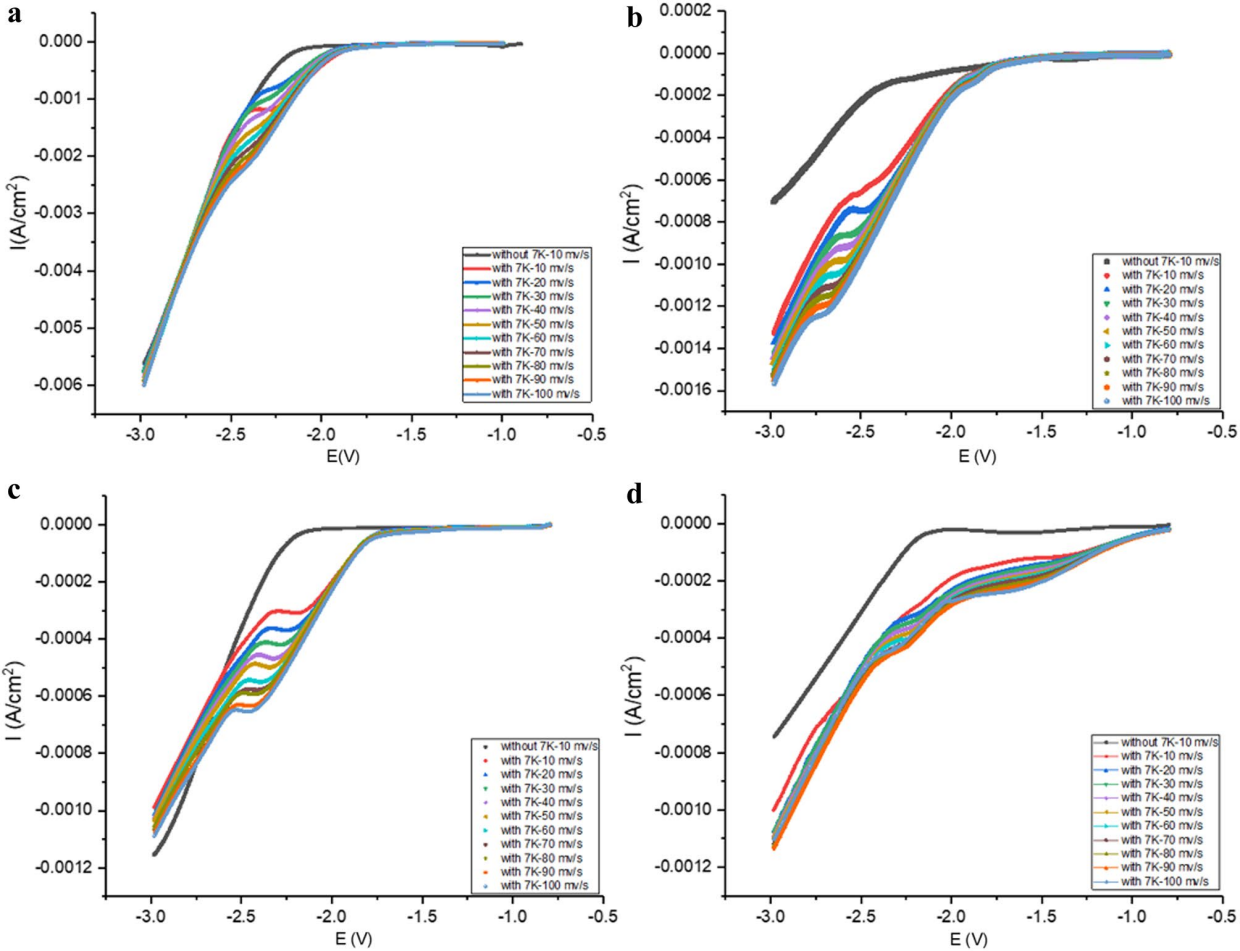
LSV of without 7K-LCA at 10 mv/s of scan rate and with 7K-LCA at different scan rates (**a**: DMF, **b**: HMPA, **c**: DMPU, **d**: DMI).

It can be seen from the Fig. 4 that in the LSV results of the electrolyte without 7K-LCA, there was no reduction peak. While in the electrolyte containing 7K-LCA, a reduction peak appeared.

**Figure 5:**
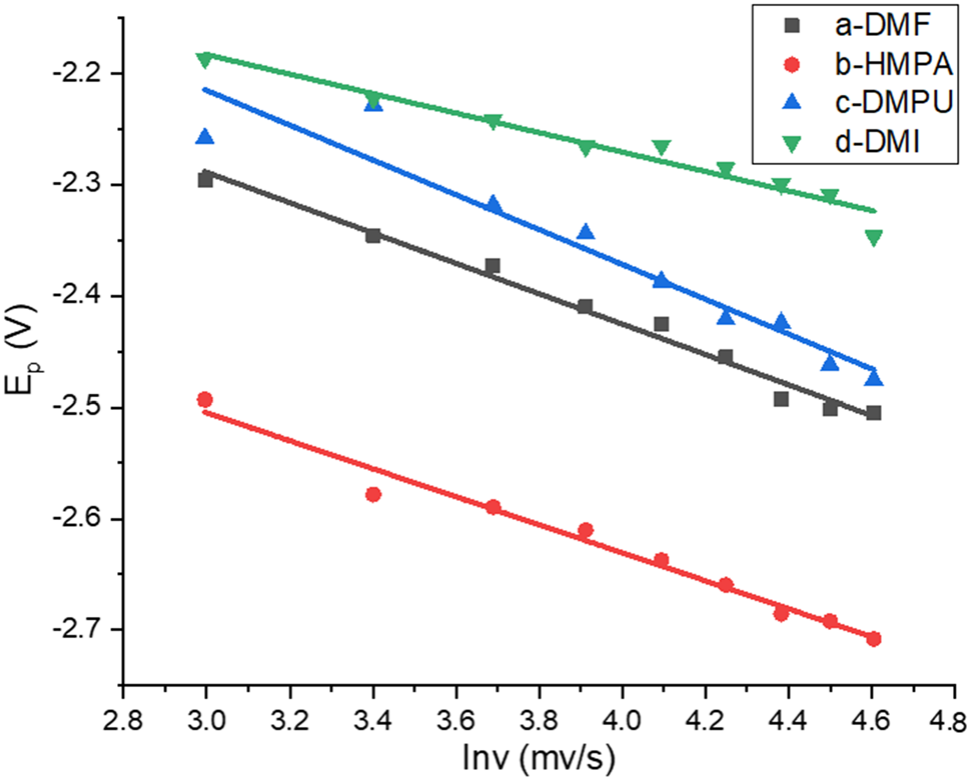
Plot of E_p_ versus lnv for DMF, HMPA, DMPU and DMI systems.

In the electrolyte containing 7K-LCA, increase the scan rate in the LSV test, it can be seen that as the scan rate increases, the reduction potential decreases negatively. As the scan rate v increases, the reduction peak E_p_ of 7K-LCA gradually shifts negatively, and the peak current i_p_ increases accordingly. Taking the reduction peak E_p_ as the ordinate and the logarithm of the different scan rate as the abscissa, the curve E_p_-lnv for DMI, HMPA, DMPU and DMI systems were shown in the Fig. 5. As shown, a straight line can be obtained, respectively, proving that the reduction of 7-ketolithcholic acid was an irreversible reaction in DMI, HMPA, DMPU or DMI. Therefore, the product obtained after the reduction of 7K-LCA will not be oxidized back to 7K-LCA, and it will not affect the conversion rate of 7K-LCA and the yield of the product by being oxidized again.

**Figure 6:**
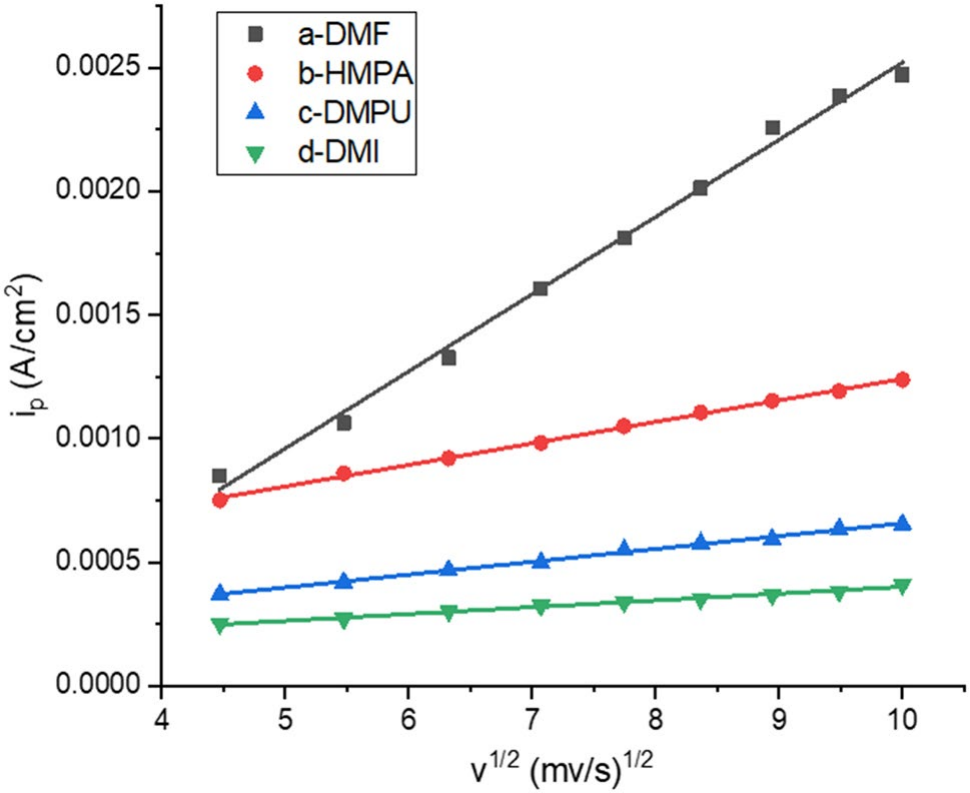
Plot i_p_ versus v^1/2^ for DMF, HMPA, DMPU and DMI systems.

Taking the reduction peak current i_p_ as the ordinate and the square root of the different scan rates as the abscissa to make the curve i_p_–v^1/2^ for DMI, HMPA, DMPU and DMI system, as shown in Fig. 6, respectively. Straight lines were obtained with a correlation coefficient of 0.9939, 0.9970, 0.9983 and 0.9890, respectively, which shows that the linear relationship between i_p_ and v^1/2^ better, it was proved that the irreversible reduction reactions was controlled by diffusion. Since the reaction was controlled by diffusion, the rotor was added to the electrolyte and placed on a magnetic stirrer. In the subsequent electrolysis process, stirring was carried out while electrolysis.

**Figure 7:**
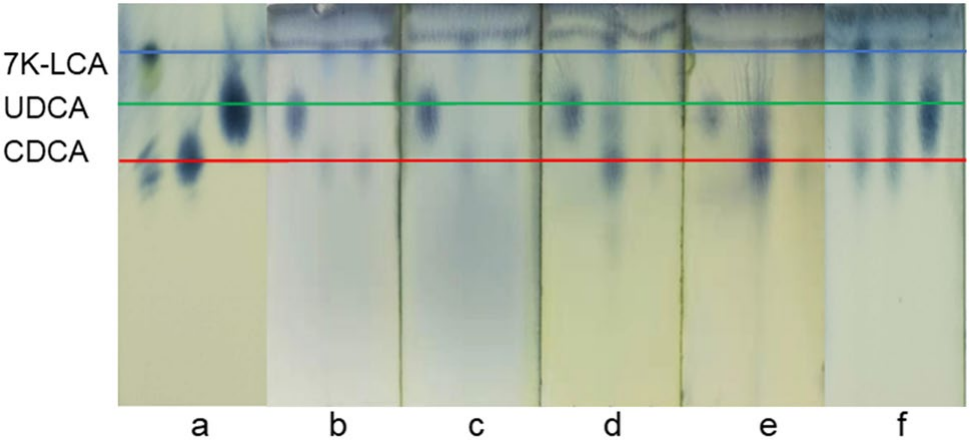
Thin plate chromatography of products and pure products.

As shown in Fig. 7a, from left to right were the thin plate chromatography results of 7K-LCA, CDCA and UDCA.

*N'N*-dimethylformamide (DMF) was used as the electrolyte, samples were taken at the electrolysis process and analyzed by HPLC. The analysis results showed that no target product was formed. After electrolysis the products in the electrolyte were separated, comparison with pure UDCA and 7K-LCA by thin plate chromatography analysis. As shown in Fig. 7 b, there was no corresponding product in sample. According to the linear sweep voltammetry results, it can be seen that as the scan rate increases, the signal of the reduction peak becomes weaker and weaker. After multiple scans, the reduction peak completely disappeared, indicated that 7K-LCA cannot be continuously reduced in DMF, this was consistent with the results of product detection after electrolysis.

Dimethyl sulfoxide (DMSO) was used as the electrolyte, samples were taken at the electrolysis process and analyzed by HPLC. The results showed that the content of 7K-LCA did not change. However, an absorption peak of a new substance appeared in the liquid phase spectrum, and with the progress of electrolysis, the peak area of the absorption peak of 7K-LCA remained unchanged, the area of the absorption peak of the new substance became larger and larger. This new substance was considered to be a new substance generated by the reduction reaction of the solvent during the electrolysis process, this result was consistent with the result obtained by linear sweep voltammetry. The thin plate chromatography method was also used to compare the separated sample with the pure products of UDCA and 7K-LCA. As shown in Fig. 7c, also confirmed that no UDCA was generated.

Hexamethylphosphoric triamide (HMPA) was used as the electrolyte, samples were taken at the electrolysis process and analyzed by HPLC. The analysis found that the content of 7K-LCA decreased significantly with the progress of electrolysis. It also shows that UDCA was generated, and as the electrolysis continues, the absorption peak area becomes larger and larger, The electrolysis continues until the liquid phase analysis of the electrolyte sample taken has no 7K-LCA absorption peak. With the increased of electrolysis time, the conversion rate of 7K-LCA and the yield of UDCA were listed in Table 1. HPLC analysis showed that the retention time of HMPA was the same as that of CDCA. Therefore, the high performance liquid chromatography analysis of the sample during the electrolysis process cannot calculate the yield of CDCA. After the end of electrolysis, the product was isolated, re-dissolved in methanol, and compared with pure CDCA by HPLC analysis. It was found that the final product contained CDCA, the standard curve prepared by dissolving CDCA in methanol was used to calculate the yield of CDCA. After separated the electrolyte and product, the conversion rate of 7K-LCA, yield of UDCA and yield of CDCA were listed in Table 2. The properties of the product were determined again by thin plate chromatography, as shown in Fig. 7d. Comparing the product with the UDCA and the substrate 7K-LCA can also see that the UDCA sample point appears, indicated that the product contains UDCA. Since the purity of the substrate contains a small amount of CDCA, the substrate has CDCA sample spots. After comparison, it was found that the CDCA sample spots in the product were darker than the CDCA sample spots of the substrate. It can also prove that the concentration of CDCA maybe increased.

**Table 1:** 7K-LCA conversion rate and product yield during electrolysis.

Solvent	Charge /C	C (%)	Y_u_ (%)	Y_c_ (%)
HMPA	720	36.7	5.8	–
DMPU		30.5	15.5	16.5
DMI		26.0	12.4	–
HMPA	1440	67.1	11.6	–
DMPU		37.5	14.6	27.4
DMI		39.0	19.7	–
HMPA	2880	84.8	20.4	–
DMPU		41.4	18.0	46.3
DMI		56.3	30.0	–
HMPA	4320	99.8	24.1	–
DMPU		42.0	11.8	71.4
DMI		56.7	33.3

**Table 2:** Conversion rate of 7K-LCA and yield of product after electrolysis.

Solvents	C (%)	Y_u_ (%)	Y_c_ (%)	ee (%)
DMF	–	–	–	–
DMSO	–	–	–	–
HMPA	97.4 ± 1.36	18.9 ± 1.28	32.0 ± 1.41	− 25.7
DMPU	40.6 ± 1.25	10.4 ± 1.12	24.3 ± 1.09	− 40.1
DMI	55.6 ± 1.16	30.5 ± 1.03	–	100

Later, considering that the safety risk of using hexamethylphosphoric triamide as the electrolyte was high, We found similar aprotic polar solvents 1,3-dimethyl propylene urea and 1,3-dimethyl-2- imidazolinone were used to replace hexamethylphosphoric triamide. Due to the relative safety of DMPU and DMI, DMPU and DMI were often used as solvents instead of HMPA in other studies.

When 1,3-dimethyl propylene urea (DMPU) as the electrolyte, the HPLC analysis was performed on the samples during and after the electrolysis. The analysis results found that the content of 7K-LCA decreased and UDCA increased significantly with the progress of electrolysis. The analysis also showed that the sample contained CDCA and increased as the electrolysis. With the increased of electrolysis time, the conversion rate of 7K-LCA and the yield of UDCA were listed in Table 1. The time of electrolysis was the same as that of HMPA as the solvent, it was found that 7K-LCA cannot be completely converted, both UDCA and CDCA yields were lower than when HMPA was used as a solvent. The thin plate chromatography method was used to determine the properties of the product again, as shown in Fig. 7 e, which also proved that UDCA was generated.

When 1,3-dimethyl-2-imidazolidinone (DMI) as the electrolyte, samples were taken at the electrolysis process and end of the electrolysis, analyzed by high performance liquid chromatography. The analysis found that with the progress of electrolysis, the concentration of 7K-LCA decreased significantly, the concentration of UDCA increased significantly. It also showed that no CDCA was generated. Similarly, thin layer chromatography was used to determine the composition of the product again, as shown in Fig. 7f, which proved that UDCA was generated.

In order to calculate the yield of CDCA with HMPA as the electrolyte. Precipitation the product and re-dissolve it in methanol, and the yield of the corresponding product was calculated by high performance liquid chromatography. In several other solvents, the products were also separated after electrolysis, and then dissolved in methanol to calculate the corresponding conversion rate or yield, listed in Table 2.

DMSO and DMF as electrolyte, respectively, no corresponding product was obtained after electrolysis. When HMPA and DMPU as electrolyte, respectively, both UDCA and CDCA were detected at the end of electrolysis. DMI as electrolyte, there was no CDCA was detected in electrolyte, so electrolysis in DMI shows stereoselective reduction of 7K-LCA to UDCA.

Regarding why stereo selectivity was only shown in DMI, we make the following explanation. Due to the configuration effect of DMI, the dipole was reduced and the carbonyl reaction activity was reduced, and the plane structure of the imidazole five-membered ring reduces the chance of the carbonyl group being attacked, so its structure was stable, and HMPA and DMPU are easily attacked. The structure was easily destroyed. The imidazole structure of DMI also makes it more basic than HMPA and DMPU.

**Figure 8:**
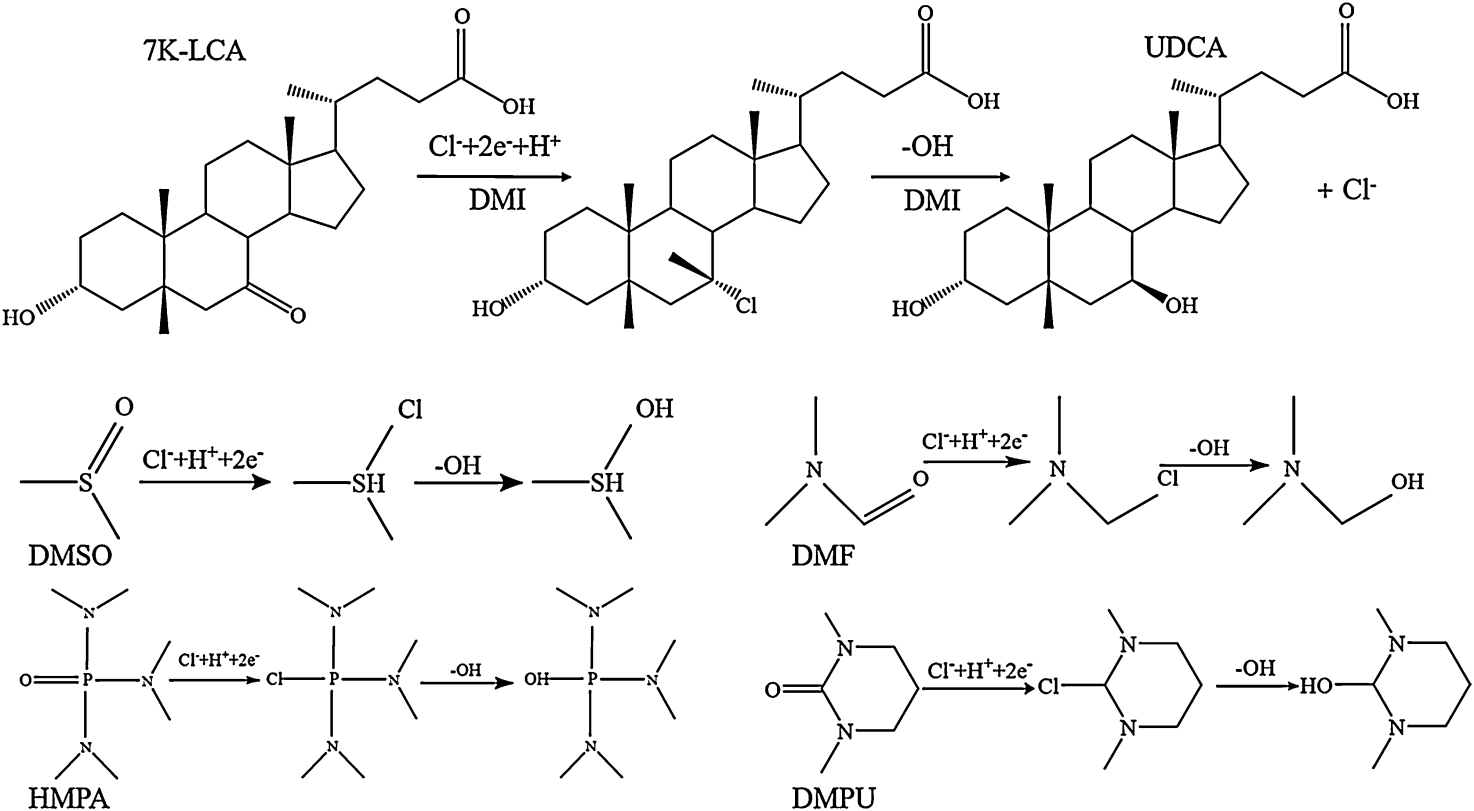
Schematic diagram of reaction mechanism in DMSO, DMF, DMPU, HMPA and DMI.

There are many alkyl groups connected to the 7-position carbonyl carbon atom of 7K, which weakens its positive charge. However, by increasing the basicity of the system, the nucleophilicity of the nucleophile can be increased. In DMI, lithium chloride acts as a nucleophile. Due to the strong alkalinity of the system, it was transformed into chloride ion with stronger nucleophilicity, and the nucleophilicity of chloride ion increases, and a nucleophilic substitution reaction occurs. Chloride ion attacks the carbonyl group at position 7 to break the carbon–oxygen double bond. The carbon at position 7 connects with chloride ion and obtains a proton. Due to the large steric hindrance of the β configuration, the configuration at the 7 position was α configuration. The removed oxygen ions combine with hydrogen ions to obtain -OH. Since the hydroxyl group was more nucleophilic, the 7-position carbon was attacked by the hydroxyl group again, causing the chloride ion to leave, and the hydroxyl group was connected to the 7-position carbon. The bimolecular nucleophilic reaction causes the configuration of position 7 to be reversed, thus only UDCA was formed (Fig. 8). In HMPA and DMPU, only a simple reduction reaction occurs, so two configurations of products are obtained by reduction, and because the steric hindrance of the formation of CDCA was small, the amount of CDCA generated was more than that of UDCA.

Similarly, the linear voltammogram obtained in the DMI test was also reflected, and two current fluctuations occurred during the scanning process, indicating that two nucleophilic reactions occurred. In HMPA and DMPU, only a reduction reaction occurred, and only one current fluctuation occurred during the scanning process.

### Effect of different concentrations of supporting electrolyte on electroreduction

After the electrolysis, the conversion rate of 7K-LCA, yield of UDCA and CDCA were calculated, respectively. Listed in the Table 3.

**Table 3:** Influence of electrolyte concentrations on the stereoselective electroreduction of 7K-LCA.

Solvents	LiCl	C (%)	Y_u_ (%)	Y_c_ (%)	ee (%)
HMPA	0.2 M	78.7 ± 1.33	9.8 ± 1.21	24.3 ± 1.40	− 42.5
	0.3 M	–	–	–	–
DMPU	0.2 M	49.8 ± 1.32	9.9 ± 1.12	10.3 ± 1.06	− 2.0
	0.3 M	64.7 ± 1.27	9.8 ± 1.18	19.6 ± 1.09	− 33.3
DMI	0.2 M	19.2 ± 1.02	13.4 ± 1.14	–	100
	0.3 M	10.3 ± 1.10	6.7 ± 1.01	–	100

After increasing the concentration of LiCl, from the above results, the conversion rate of the substrate and the yield of the product both decrease. In the above electrolytic system, because the ion concentration was too high, the ion conductivity does not increase but decreases instead. The dielectric constant of these three solvents was lower than that of water. the degree of ionization decreases. Since the dielectric constant of HMPA was relatively low, 0.75 g of LiCl cannot be completely dissolved, so there was no corresponding data.

### Effects of different cathode electrode materials on electroreduction

Therefore, based on the research of 3.1 and 3.2, when DMI was selected as the electrolyte, the analysis result of the product of the electrolytic reaction showed that no by-product CDCA was generated, so that the separation step of UDCA and CDCA could be ignored. Therefore, when the electrolysis in DMI was carried out, the effect of replacing mercury-plated copper with lead on the yield of reduced products was studied.

The 7K-LCA conversion rate, UDCA yield and CDCA yield obtained by using lead plates and mercury plated copper plates as cathode electrodes corresponding to different concentrations of supporting electrolyte in the previous section were listed in the Table 4.

**Table 4:** Influence of cathode materials on the stereoselective electroreduction of 7K-LCA.

Solvent	LiCl	Electrode	C (%)	Y_u_ (%)	Y_c_ (%)
DMI	0.1 M	Hg–Cu	55.6 ± 1.16	30.5 ± 1.03	–
	0.2 M	Hg–Cu	19.2 ± 1.02	13.4 ± 1.14	–
	0.3 M	Hg–Cu	10.3 ± 1.10	6.7 ± 1.01	–
	0.1 M	Pb	48.2 ± 0.98	16.6 ± 1.02	–
	0.2 M	Pb	58.3 ± 1.02	34.9 ± 1.12	–
	0.3 M	Pb	19.1 ± 0.99	10.6 ± 1.03	–

Since the resistivity of mercury was as high as 984 nΩ·m, and the resistivity of lead was 206.84 nΩ·m, the lead conductivity was better than mercury. Therefore, after increasing the concentration of LiCl, the ion conductivity was increase, so the conversion rate of 7K-LCA was improved, and the yield of UDCA was also followed increase. Also, no component of CDCA was detected in the product. When the ion concentration was increased to 0.3 M, the ion conductivity decreases, the conversion rate of the substrate and the yield of the product were reduced.

For the problem that the conversion rate of the substrate was not equal to the yield of the product, Since there was a hydroxyl group at the three position of 7K-LCA, there was a keto group at the 7 position. So both oxidation and reduction reactions can occur. It was due to the oxidation of 7K-LCA at the anode to produce 3,7-diketocholanic acid, And the oxidation reaction occurs first and then provides hydrogen ions for reduction, at the same time, it can be found that the pH of the solution decreases first and then increases with electrolysis. The electrolyte was analysis by high performance liquid chromatography in the electrolysis process, it can be seen that there were absorption peaks of oxidation products. The electrolytic product can be purified by silylation crystallization to isolate ursodeoxycholic acid. In order to control the oxidation amount of the substrate, other organic reagents as hydrogen ion donors can be added in the subsequent research.

### Characterization of product

**Figure 9:**
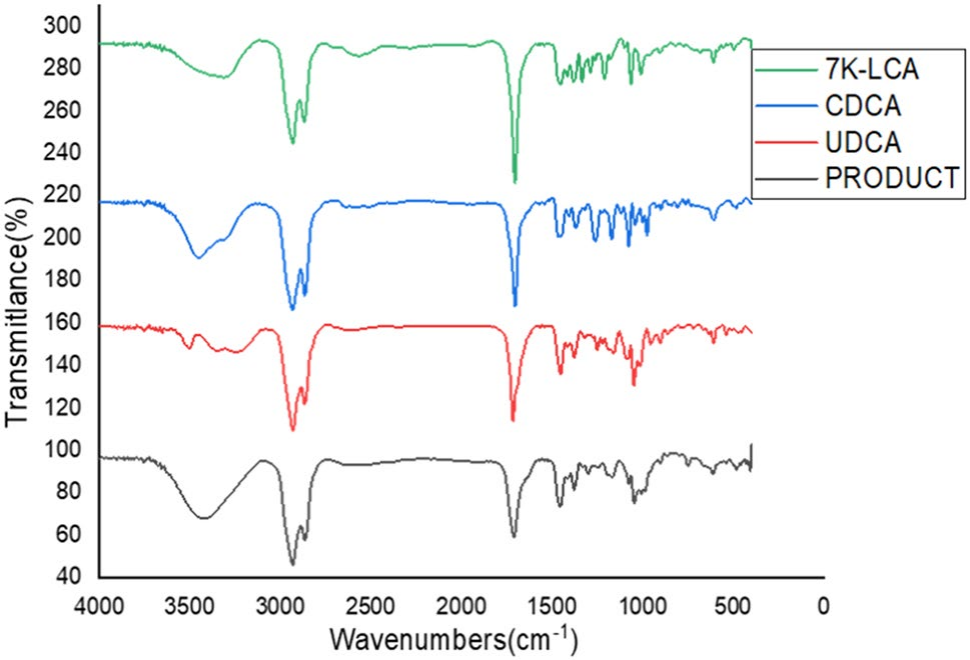
Comparison of infrared spectrum of product and related pure products.

**Figure 10:**
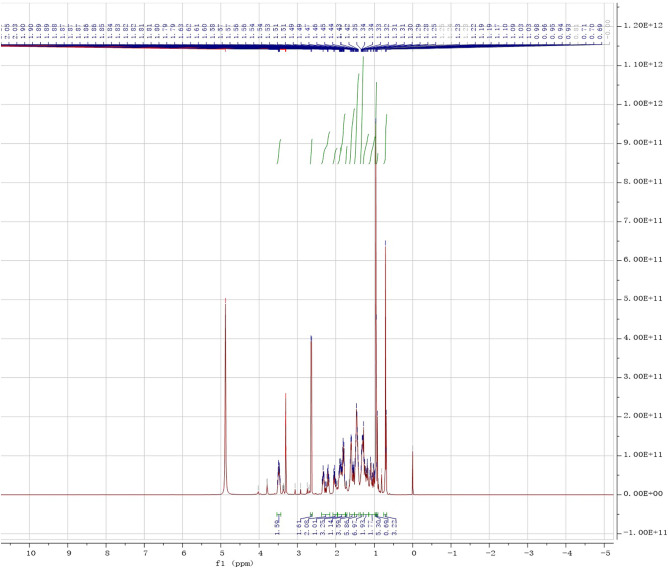
^1^H NMR spectrum of the product.

**Figure 11:**
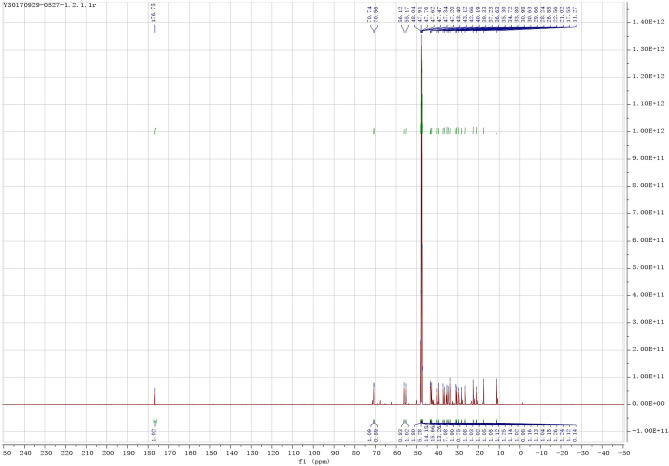
^13^C NMR spectrum of the product.

The product was characterized by using IR, ^1^H NMR and ^13^C NMR. As was shown in Figs. 9, 10 and 11, respectively. The IR spectrum demonstrated that the (CH_3_)_2_CH– or (CH_3_)_3_C– (1380 cm^−1^), –C–OH (1050 cm^−1^), –CH– (2750–3000 cm^−1^) and –COOH (1700 cm^−1^) groups exist in the chemical structure of the product. Compared with the infrared chromatograms of UDCA, CDCA and 7K-LCA pure products, UDCA has an outward hydroxyl characteristic group (−C–OH, 1050 cm^−1^), and the product also has an infrared absorption peak here, the product was UDCA.

It was known that in the hydrogen spectrum (^1^H NMR, Fig. 10), the chemical shift of hydrogen in the main group of the product were δ(2H, m, C3-H, C7-H) = 3.51, δ(1H, t, C23-H) = 3.30, δ (3H, d, C20-CH3) = 0.97, δ(3H, s, C10-CH3) = 0.96, δ(3H, s, C13-CH3) = 0.71.

It was known that in the carbon spectrum (^13^C NMR, Fig. 11), the chemical shift of carbon in the main group of the product were δ(C24-OOH) = 176.76, δ(C7-OH) = 70.56, δ(C3-OH) = 56.12, δ(C19) = 17.54, δ(C18) = 11.27.

From the above spectrum we can conclude that the product was UDCA.

## Conclusion

In previous studies, we found that electrochemical reduction in lower alcohols will cause esterification side reactions. In order to prevent the substrate from turning to non-target substances, in the research of this article, we selected five kinds of reagents with certain catalysis and stereoselectivity for the reduction reaction based on the research of other scholars. Based on the results, we found that electrolysis in DMF and DMSO did not get any products, while electrolysis in HMPA, DMPU and DMI showed stereo selectivity. In HMPA or DMPU, since the steric hindrance of CDCA was small, 7K-LCA was mainly selectively reduced to CDCA. When DMI was used as a solvent, it provides conditions for the occurrence of nucleophilic reaction. When the second nucleophilic reaction occurs, the configuration undergoes "Walden inversion", thus achieving the stereoselective reduction of 7K-LCA. it was strongly stereoselectively reduced to UDCA.

In HMPA, mercury-plated copper as the cathode, the LiCl concentration was 0.1 M, the conversion rate of 7K-LCA was up to 97.4%, the yield of CDCA was 32%, the ee value was 25.7%. The LiCl concentration was 0.2 M, the ee value was 42.5%. In DMPU, mercury-plated copper as the cathode, the LiCl concentration was 0.1 M, the conversion rate of 7K-LCA was 40.6%, the yield of CDCA was 24.3%, the ee value was 40.1%, and the conversion rate of 7K-LCA increases as the concentration of LiCl increases.

When DMI as electrolyte, mercury-plated copper as the cathode, the LiCl concentration was 0.1 M, the conversion rate of 7K-LCA was 55.6%, the yield of UDCA was 30.5%. Lead as the cathode, the LiCl concentration was 0.2 M. the conversion rate of 7K-LCA was 58.3%, the yield of UDCA was 34.9%. No matter under any conditions, with DMI as the electrolyte, the ee value was 100%. IR, ^1^H NMR and ^13^C NMR characterizations proved that the product was UDCA.

Compared with chemical and biological preparative processes, the electrochemical process has advantages of low cost, stable response and simple steps. Compared with the separated electrolytic cell, the electrochemical reduction of ursodeoxycholic acid in the undivided electrolytic cell further simplifies the process steps, avoids the esterification side reaction, and improves the conversion rate of the substrate into the target product. At the same time, the stereoselective production of the target product was achieved in the research of this article.
